# Supplementary material for: Mobile element insertions and associated structural variants in longitudinal breast cancer samples
Source: Sci Rep. 2021 Jun 22;11:13020. doi: 10.1038/s41598-021-92444-0 (PMC8219704; doi:10.1038/s41598-021-92444-0)
Supplement: Supplementary file 1 — Supplementary Informations. [file 41598_2021_92444_MOESM1_ESM.pdf]

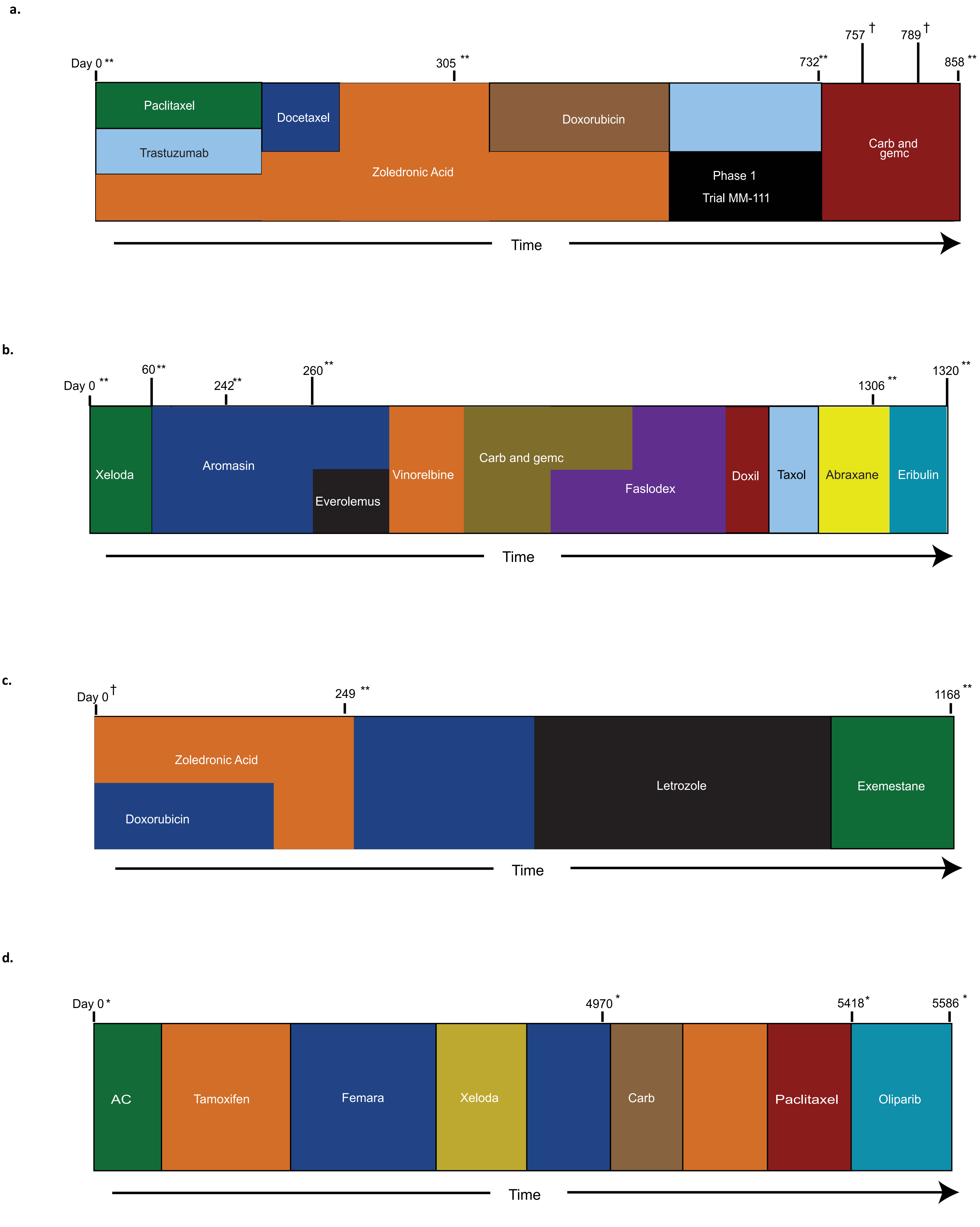

Supplemental Figure 1. Outline of patient treatment information. Parts A-D correspond with patients 1-4, respectively. Each medication or treatment is identified by a colored box. If a treatment was used more than once, a box of the same color is used without a label in that patient to represent the same treatment. The length of the box approximates the duration of each treatment. If multiple treatments overlapped, the boxes are stacked through the timepoints for which they overlapped. The timepoint of each sampling event is shown on the top of the box in days after the first sampling timepoint. *= DNA, **= DNA and RNA, †= RNA

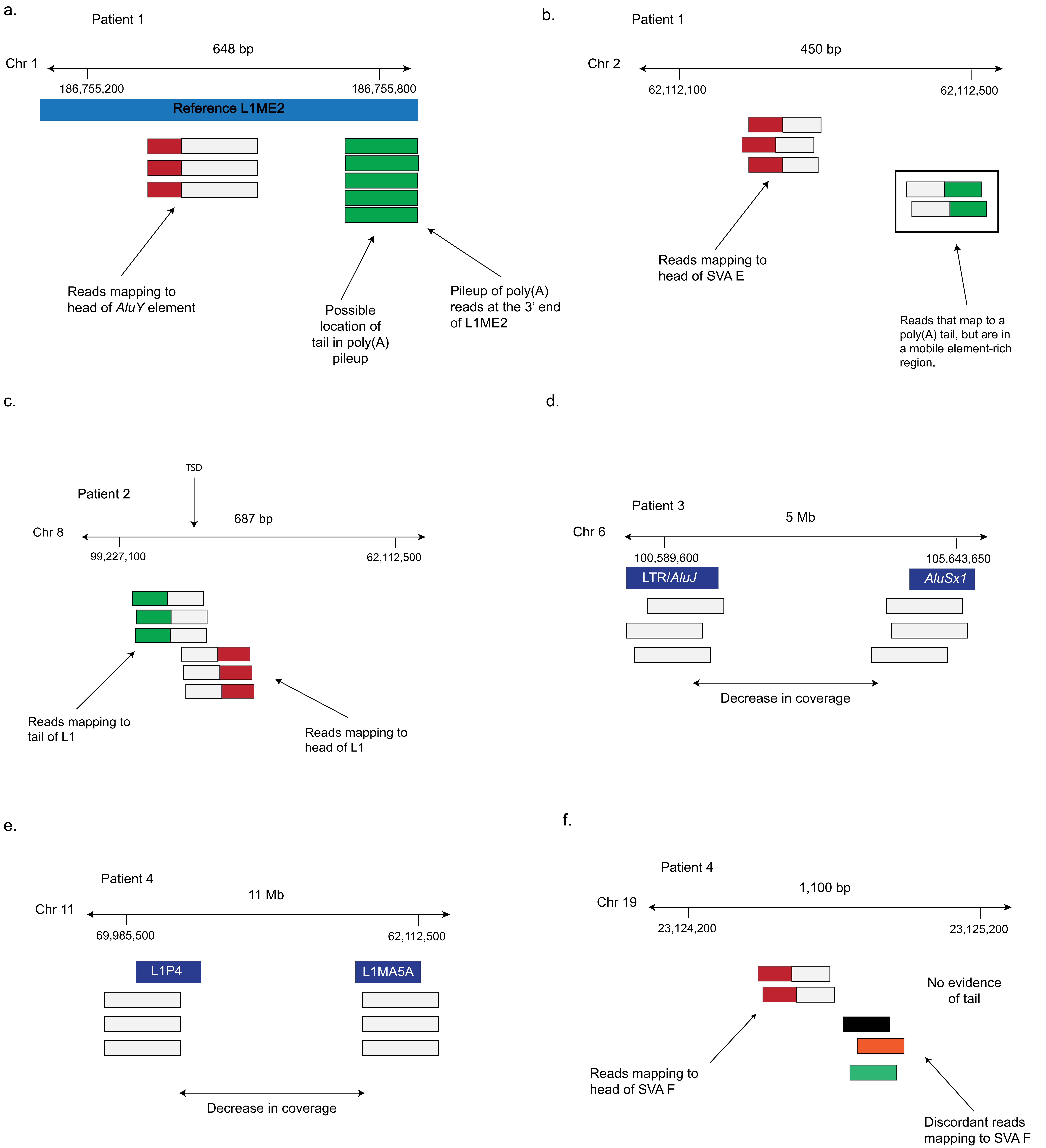

Supplemental Figure 2. Schematics of the remaining mobile element insertions and associated structural variants identified in this project. All mobile element subfamilies were determined using the UCSC genome browser for reference insertions and RepeatMasker for somatic insertions. A) Insertion of an *Alu* element inside an L1ME2. The insertion appears to be a non-classical insertion as there is no direct evidence of a poly(A) tail. It is possible that these reads have mapped to the poly(A) tail of the L1. B) Insertion of an SVA E in Patient 1. Example reads that map to the head and tail of the element are shown below the figure. Few reads line up to a potential tail of the insertion. C) L1 insertion in Patient 2. Example reads that map to the head and tail of the L1 insertions are shown below the figure. The location of the target site duplication (TSD) is shown above the reads. D) A mobile element mediated deletion in Patient 3. This appears to be a complex rearrangement between an *AluSx1* and a rearrangment that had previously occurred. E) A mobile element mediated deletion in Patient 4 between two reference L1 insertions. A decrease in coverage is visible between the two elements. F) An SVA F insertion in Patient 4. Reads mapping to the head of the SVA F were visible, but the reads mapping to the tail of the insertion were not found. There were multiple discordant reads just downstream of the insertion site that mapped to the SVA F. This may be a non-classical insertion or the insertion may be present at too low of a frequency to accurately identify the tail.

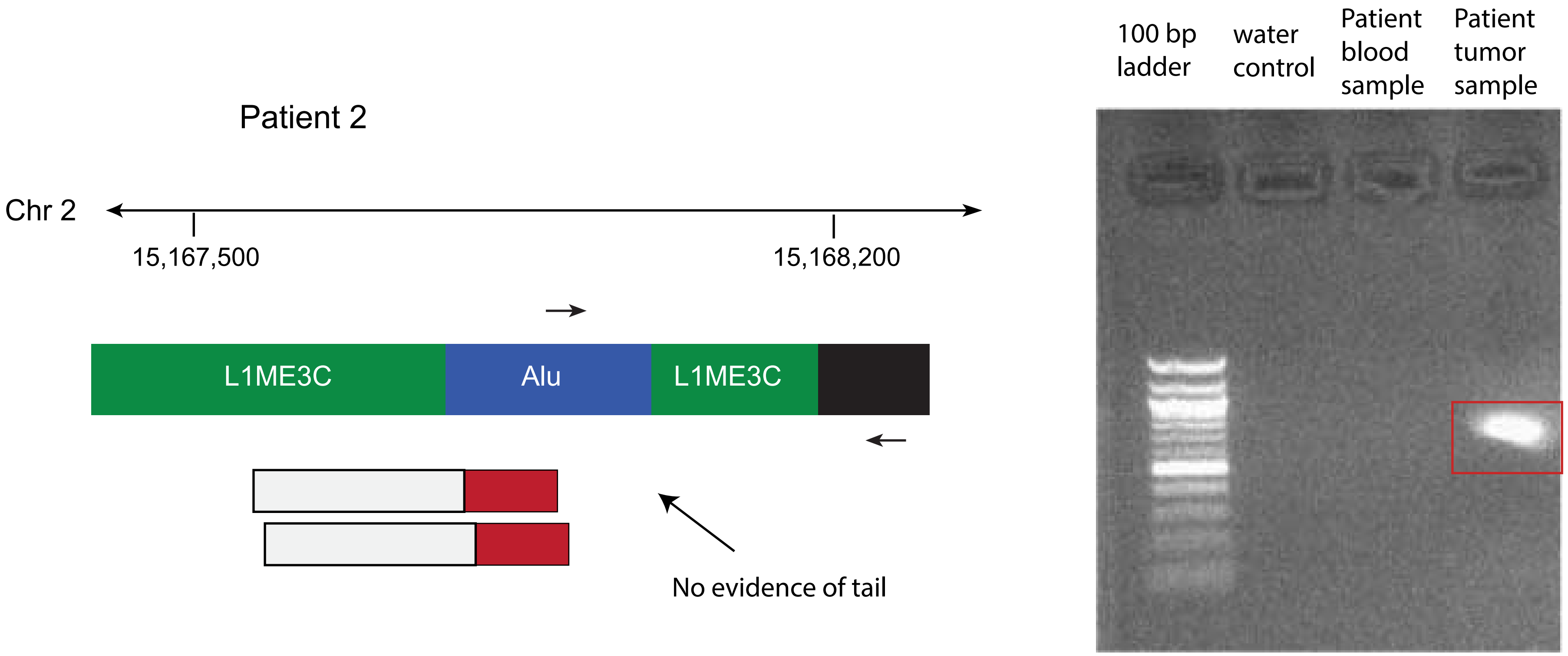

Supplemental Figure 3. PCR validation of an Alu element from Patient 2. A schematic of primer design is shown on the left, with one external primer in unique genomic DNA, and one internal primer in the *Alu* element. An example of sequencing reads mapping to the head of the *Alu* insertion are shown as red and white boxes. This appears to be a non-classical insertion with no evidence of the tail of the element in the sequencing reads. A red box is surrounding the band of interest in the tumor DNA, which is not present in the matched germline DNA sample.

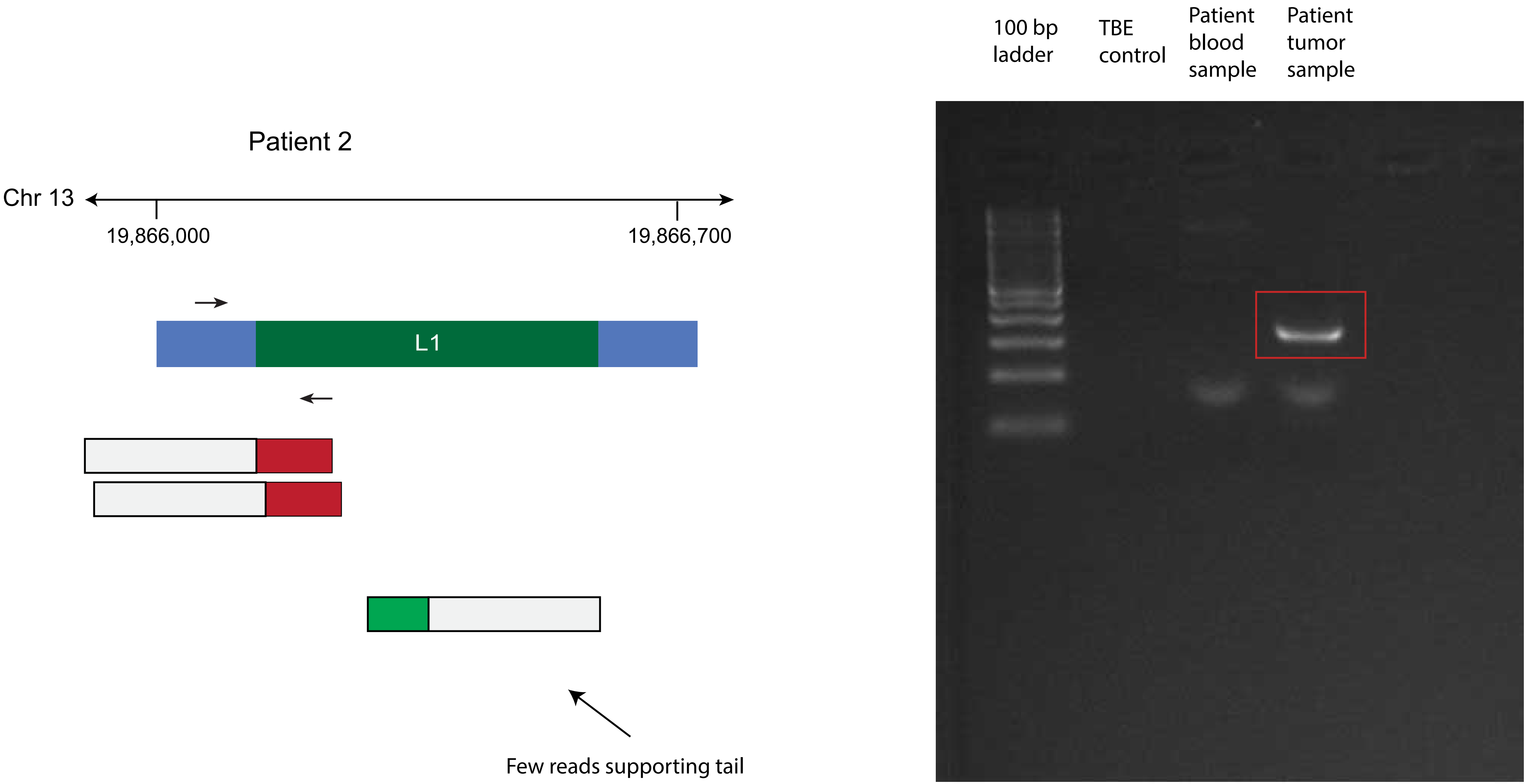

Supplemental Figure 4. PCR validation of a L1 from Patient 2. A schematic of primer design is shown on the left, with one external primer in unique genomic DNA, and one internal primer in the L1. An example of sequencing reads mapping to the head of the L1 insertion are shown as red and white boxes while the reads mapping to the tail of element are shown as green and white. A red box is surrounding the band of interest in the tumor DNA, which is not present in the matched germline DNA sample.

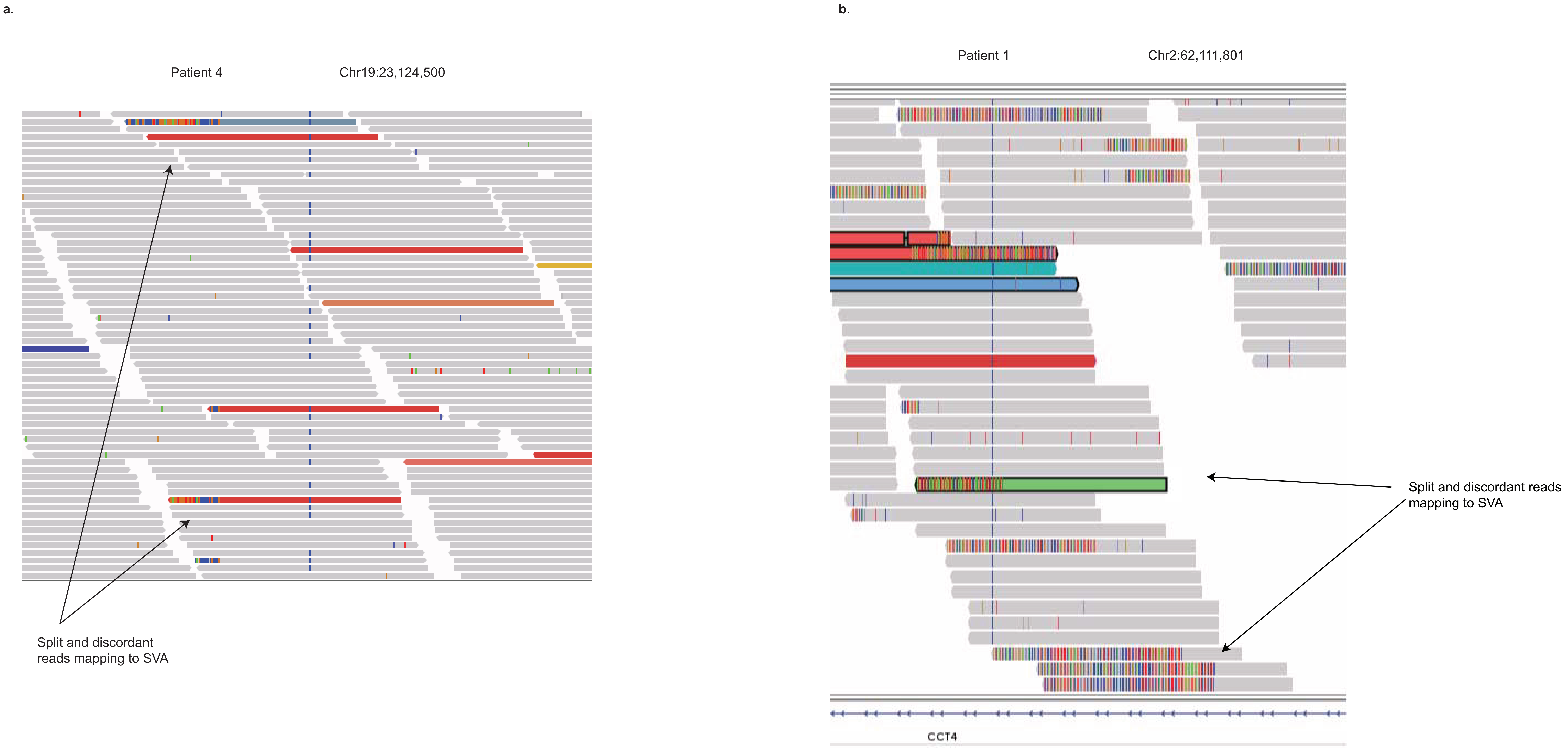

Supplemental Figure 5. IGV images showing the split and discordant reads that map to the two SVA insertions identified in this study. Part a) shows the insertion in Patient 4, while b) shows the insertion in a mobile element-rich region in Patient 1. Some of the discordant or split reads mapping to each insertion are denoted with arrows.

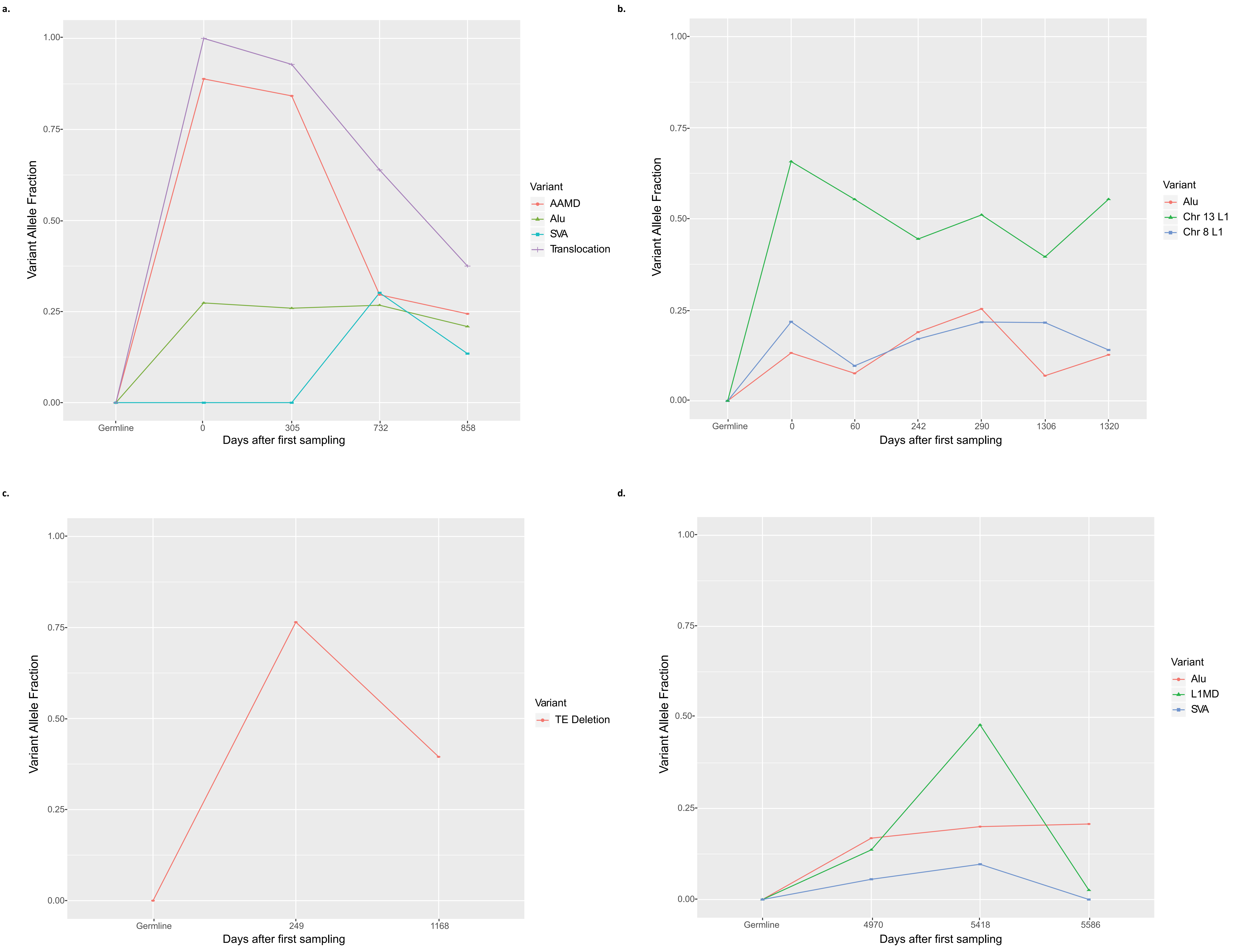

Supplemental Figure 6. Unadjusted variant allele fraction (VAF) for each of the four patients. Patients 1-4 are shown in parts A-D, respectively. VAF is shown on the Y axis, with the sampling timepoints for each patient shown on the X axis.

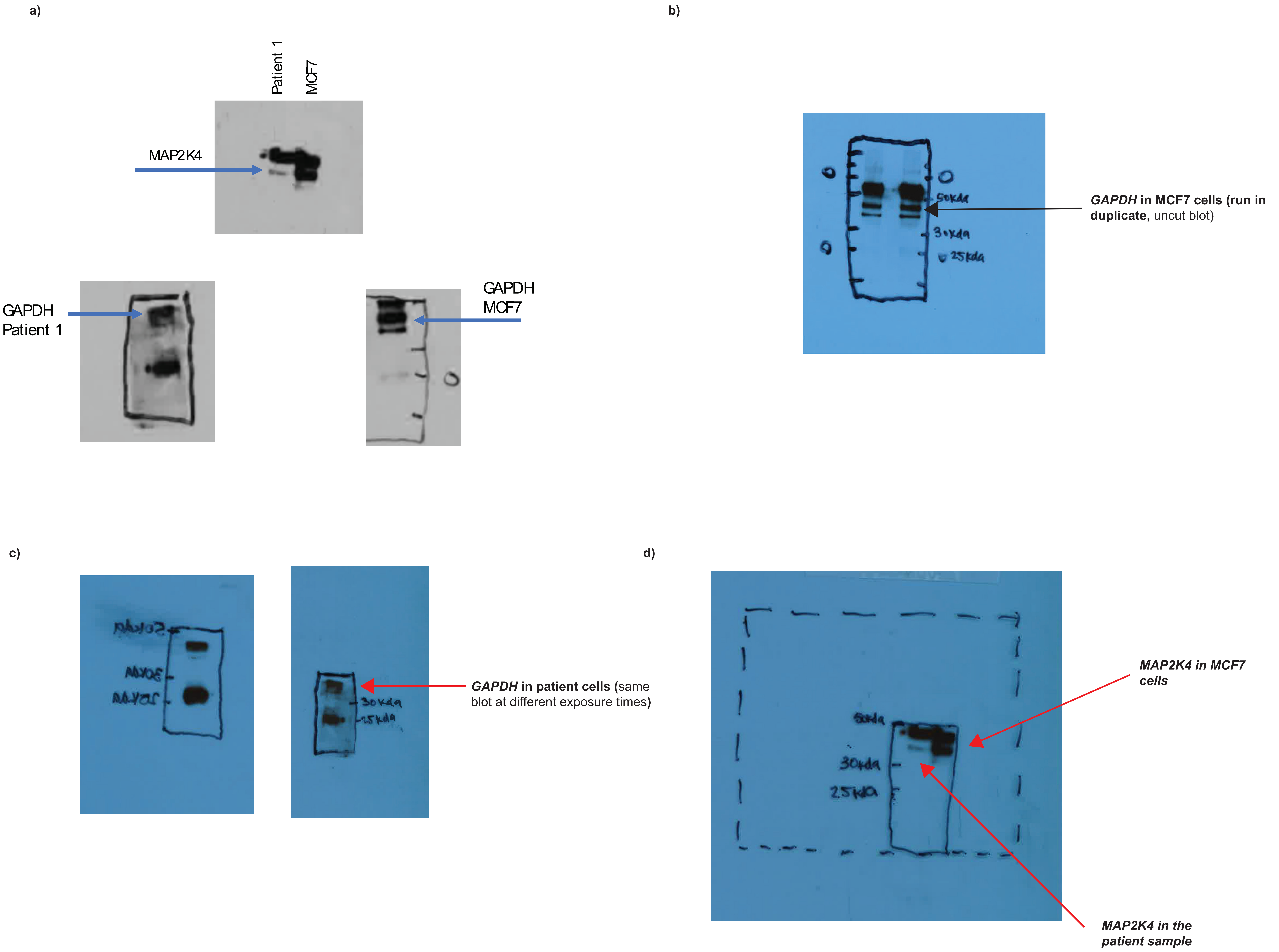

Supplemental Figure 7. Uncropped Western blot images for Patient 1 and MCF7 cells. Images were produced on X-ray film and do not show edges of an actual, full membrane. Blots were cut post-antibody incubation to ensure a clear image of the blots; molecular marker illustrated with sharpie. a) MAP2K4 is shown in the top portion of the figure, while the loading control GAPDH is shown in the bottom portion of the figure. b) Duplicate runs of GAPDH in MCF7 cells on membrane without top portion cut. c) Blot of GAPDH in the cells from Patient 1 at different exposure times. d) Western blot of MAP2K4 in the patient sample (left lane) and MCF7 cells (right lane) with size of full membrane outlined with a dashed line.

Supplemental Table S1. Tools that identified each insertion or structural variant. An "X" denotes that the tool identified the variant. RUFUS was unable to run on Patient 4. This has been denoted with "N/A".

**Patient**	**Locus**	**Variant**	**MELT**	**TranSurVeyor**	**RUFUS**
Patient 1	1:186,755,400	*Alu* (non-classical insertion)	X		X
	1:29,089,030	*Alu-Alu* associated deletion (23kb)	X	*X*	X
	2:62,111,770	SVA insertion	X		X
	17:11,974,341/ 22:48,343,831	*Alu* -associated translocation	X	*X*	X
Patient 2	2:15,168,000	*Alu* (non-classical insertion)	X	X	X
	8:99,227,400	L1 insertion	X	*X*	X
	13:19,866,150	L1 insertion		*X*	
Patient 3	6:105,643,750	*Alu-Alu* associated deletion	X	X	X
Patient 4	10:67,431,020	*Alu* insertion	X	X	N/A
	11:81,149,160	L1-associated deletion	X	X	N/A
	19:23,124,500	SVA insertion	X		N/A

Supplemental Table S2. Estimates of tumor purity, average genome-wide copy number, and genome-wide coverage (sequencing depth) for each of the samples at each timepoint.

Patient	Sampling timepoint	Estimated Tumor Purity (%)	Genomic Copy Number	Genome-wide Coverage
Patient 1	Germline	-	-	74.63
	Day 0	94.5	1.88	81.87
	Day 305	90.75	1.87	74.2
	Day 732	79.21	1.86	73.04
	Day 858	60.76	1.86	74.23
Patient 2	Germline	-	-	32
	Day 0	89.62	3.9	70.51
	Day 60	88.51	3.98	73.03
	Day 242	79.75	3.86	72.14
	Day 290	87.32	3.96	69.27
	Day 1305	78.97	3.73	67.2
	Day 1320	72.15	3.73	73.74
Patient 3	Germline	-	-	72.42
	Day 249	85.51	2.09	70.88
	Day 1168	81.82	1.95	62.24
Patient 4	Germline	-	-	29.7
	Day 0	30.91	4.12	54.78
	Day 2970	85.91	1.98	60.81
	Day 5418	74.78	2.01	58.49
	Day 5586	39.58	2.03	58.72

Supplemental Table S3. MAP2K4 transcripts produced by Trinity.

>TRINITY_DN93516_c0_g2_i1 len=235 path=[0:0-234]
CGGACGTCATCAAAGATCCCTGGGAGTCGGTTGGCCTGCGGCAGCTCGTT
GCTTTTCTTCGGCTGCCTCCTCCTCAGTGGCCTCACTGCCCGAGGGGTGC
GGATGGGACTCGGACTGGCCCTGAAGAAAGTGACCACGAAAGGCTGTTGG
GAGCGTGGGGCCCGTTGACCCAGCAGGCCGGCCAGGCCAGGATCCACGCT
GTGCCATGTGTGGGTTTTGAACTCCTGTAGGATTG

>TRINITY_DN10603_c0_g2_i1 len=247 path=[0:0-246]
GAGCCCTTTTTTTCTAGAGTCTGATGTAGCAGCGATATCAATCGACATAC
ATGGGAGAGCTGGGAGTAGCTGGCATTTGATCCAGGATTTTACAAACATA
GCATGCGACCTCAACGGCACGTTCTTCATACATCAAAATAAAGGGATGTT
TCAGAAGCTCTTTATACTTTGGCCTTTTGGATTCATCCTTCGTAAGGCAC
AAGTTGACAAAGTTGATGAAACTCGGGGAGAATTCCCTTTCCTCAGA

>TRINITY_DN102065_c0_g1_i1 len=1612 path=[0:0-[illegible] 2:1369-1611]
CGGCCTTTGGCTGGCTCAGAACAAAGGACCCACCTGTACCACAGCTGAGC
TGAGAGAATGGAGGCATTTGTCCTTAACTCAGAACAGTCCCCAGACTTAT
TGATCTCAGAACCAAATGTCAAATACCCCACTTTCCGAAATAATTGTCTC
TCTTGAAAGTTCTGTGCAAATTGAATTTTTATAAATCAAATTGTATTGTA
AAGCCAGTAAAGAGAAGTGCTGCTTATGGGAAGCTTGTTTGCCTTACTTT
ATAAATGGAAGCAAACCGTGCTATGAATAAGAGCTGTTAGTTAACTTTGC
TTATAAAGCCTTTTGAAAATGTTCACTTTTTTTTTTTATAATATTTGTTT
TTTTGAAAGGGGGGTTGCCAGTTCTGCGACTGTATGTTTGGAACATCAAT
GAAGCGGGGGCCTTGAGAGTATCCGGAGTCGAAAAGGATTTCCCATTCCA
GGGAAGGGTCTGGAATAGTAAAGAAGGCATGGCTTCTAGAGTCCTCTGCT
ATCCCCTCATGACACATTGATTGAGCACAGTCGGTGCCAGCCACCTTTCC
AGTCTTGGGTCACTCCTGAGTCCAGTGGGTCTCCAGTGTGAGTGTGCACC
ACAGTCACCCGGGGGTCTATCAAAACACAGGCCTTGGTGCTCCCACCCCC
AGAGTTTCTGACTCGGTAGGTCAGGATATAGCTTGAGAATGTGCATTTCT
AGTGAGTCCTCAGGTGGTGCTGAGGCTGCTGGTTCTGACGCACTTTGAGA
ATCACTGATCGGTGAATACACGAAAGTCCTTCCCGTTCTGGGAGTTGATA
GTCTCTACAGAACCGTGCCTTCCTTTTCTGGCCCTGTGAGTCTTAGCTGG
CAATGAACACGGAAGCTGGCTTGTCTATGACGTCCCTTCGCGGTGCTGTA
GGGATTTAGTATTCAGCATGTGGAAGGTTCTCAGTAAGACTTCTTCTTAT
CTTTGAGTTTGGAAACATGATTGACAGAAATTCAGTTTGACTTGTTGGGG
TATGAATCTGTAGATGTGGGTCTGAGTTCTGATTCGGTGAGATCCGTCTG
TATAACTCAGTCATCCTTTCACTGCTTGGACATTTATTTCCTTTTCTATG
GAATGCATTCACTTCTTCATTCATTTCAACAGATAATCATAGAGCACCAA
CTCTATCTGCACCATGGATTTTGCAAGCTCCCTGCACATCACTGCAACTG
TTCTTCTGTGTCCGTACTGTTACGATCTCTAGATGAATACTGCTTTATGC
ATCCTTCTGCAATGCAGCTCTGTTAATTCCGGAAATTTCCACAGTAAACT
CGGTCTGTCAGTTACCATGGAAACTGCCTGCTTCCTGGCACTACCAACAA
ATGAGTATCTTTGCTGTGATATGTGTGGGTTTTGAACTCCTGTAGGATTG
GGATTCAGAGTAAACCTTGCTGTAGATTTGAAAGGTGGATTTGCAAAATT

CAACTTCAGTGCTTTGCGTTTACCCTGCATGCTGCTGACGGCCGGGTGGC
CTGGCGCCGGGGACCCTACGGGGCCGGGGGTGCCGCTGCCGCTGCCGCCC
CCGGAGCCGCCGCCGCCGCTCGGGCTCGGAGCCGCCATTGTTGGGAGTGA
AGAGCCGAGCGG

>TRINITY_DN102065_c0_g1_i2 len=369 path=[1:0-[illegible] 2:126-368]
AGAACCATAAGCTCCTCGTCCAATTTCTCCAAGGTCTTTCAAGTCCTCTG
CAGTGAAATCCCAGTGTTGTTCAGGGGAGATCTTCAGTTTTCCTGATGAC
TCAATGCTGTGTGTTCTCAGTCTCTCTATGTGTGGGTTTTGAACTCCTGT
AGGATTGGGATTCAGAGTAAACCTTGCTGTAGATTTGAAAGGTGGATTTG
CAAAATTCAACTTCAGTGCTTTGCGTTTACCCTGCATGCTGCTGACGGCC
GGGTGGCCTGGCGCCGGGGACCCTACGGGGCCGGGGGTGCCGCTGCCGCT
GCCGCCCCCGGAGCCGCCGCCGCCGCTCGGGCTCGGAGCCGCCATTGTTG
GGAGTGAAGAGCCGAGCGG

>TRINITY_DN97116_c0_g1_i1 len=604 path=[0:0-603]
AGATGAGGATCCGAAACGGCAGCCTTTACGTTCATCACCTGCTAGAACCT
CTCGTAGTCCATCACCATTTCTTGGCATTGGAATTCTACTGGAAAAAAAT
ACAAAAAGCAAAACAAAACCCTCAGCACTGTTACAAGAGGCCATTTAAGT
ATCTTGTGCTTCTTCACTTACCCATTAGCCAGGTTCTCATTAGGTTTTGC
TTGGGCCTCCCTGGCACTGAACCTTAGGCTTTGTATGACAGTGAAGCAGC
ACTGTGAGTGGTTCAAGCACACTGGAATATAAAACAGTCATGGCCTGAGA
TGCAGGTGATGCCATTACAGAACCAAATCGTGGCACGTATTGCTGTGTCT
CCTCTCAGAGTGACAGTCATAAATACTGTCAAACAATAAAGGGAGAATGG
TGCTGTTTAAAGTCACATCCCTGTAAATTGCAGAATTCAAAAGTGATTAT
CTCTTTGATCTACTTGCCTCATTTCCCTATCTTCTCCCCCACGGTATCCT
AAACTTTAGACTTCCCACTGTTCTGAAAGGAGACATTGCTCTATGTCTGC
CTTCGACCACAGCAAGCCATCATCCTCCATTGCTCCCGGGGACTCAAGAG
GAAT

Supplemental Table S4. Alu, L1, and SVA loci that showed significant changes in expression from early to late timepoints in Patient 2.

chr	start	end	Mobile Element	baseMean	log2FoldChange	lfcSE	stat	pvalue	padj
1	109203930	109204222	AluJb:Alu:SINE	23.46182463	-2.109950161	0.58007411	-3.637380338	0.000275425	0.01506445
1	109206011	109206314	AluSz:Alu:SINE	45.25034576	-1.830683668	0.456046741	-4.014245699	5.96E-05	0.005047421
1	109203594	109203905	AluY:Alu:SINE	43.15747907	-1.79251291	0.491600898	-3.646276717	0.000266067	0.014681215
1	108578637	108579660	L1HS:L1:LINE	81.47747476	-2.027317557	0.583551341	-3.474103157	0.000512564	0.024072006
1	197508889	197509568	L1MC3:L1:LINE	66.87760841	1.985711868	0.45036542	4.409112643	1.04E-05	0.001345511
1	108576306	108578640	L1PA2:L1:LINE	90.46863613	-1.912077293	0.545291352	-3.506524145	0.000454	0.022293839
1	108542343	108548024	L1PA6:L1:LINE	109.2501839	-2.685625968	0.58408623	-4.597995692	4.27E-06	0.000645942
1	113411875	113414694	L1PA6:L1:LINE	31.99324918	-1.963896603	0.574529716	-3.418268105	0.00063021	0.027565948
1	203313873	203314940	L1PA6:L1:LINE	59.00695805	-2.357211257	0.577779851	-4.079774075	4.51E-05	0.004057284
1	108539962	108541032	MER11C:ERVK:LTR	32.14418849	-3.537497508	0.714849322	-4.948591818	7.48E-07	0.000162124
1	238481484	238485117	MER61-int:ERV1:L	7.392621535	2.556425981	0.791281629	3.230740973	0.001234698	0.043497647
1	112956705	112956908	MIR:MIR:SINE	15.88792149	-2.306073031	0.692030763	-3.332327339	0.000861229	0.034003844
1	108535475	108536858	SVA_D:SVA:Retrop	63.56501267	-2.792407644	0.664122547	-4.204657191	2.61E-05	0.00261738
1	109176604	109177644	SVA_D:SVA:Retrop	109.94853	-2.064922364	0.609332876	-3.388824806	0.000701928	0.029425405
1	145465616	145465780	U1:snRNA:snRNA	44.92314866	-1.979201736	0.563433064	-3.512753979	0.000443488	0.021915461
2	99548467	99548746	AluJo:Alu:SINE	51.70843005	-1.915983452	0.514481021	-3.724109097	0.000196006	0.011932687
2	212372467	212372778	AluSc:Alu:SINE	68.26249316	-2.340433017	0.550853467	-4.248739741	2.15E-05	0.002325211
2	212514524	212514805	AluSc:Alu:SINE	36.87545563	-2.387346793	0.576906462	-4.138186952	3.50E-05	0.003302948
2	100001412	100001726	AluSg:Alu:SINE	14.63545048	-2.841799116	0.749867448	-3.78973527	0.000150808	0.010005592
2	99700123	99700418	AluSg:Alu:SINE	7.203128679	-2.849842401	0.826596809	-3.447681347	0.000565421	0.025779056
2	100082798	100083115	AluSp:Alu:SINE	19.73163713	-3.295948162	0.698768963	-4.716792439	2.40E-06	0.000413409
2	99824120	99824414	AluSp:Alu:SINE	15.92456613	-3.595243685	0.753108953	-4.773869267	1.81E-06	0.000337968
2	99929110	99929357	AluSp:Alu:SINE	19.42356635	-2.839421434	0.693125233	-4.096548936	4.19E-05	0.003829495
2	100062968	100063273	AluSq:Alu:SINE	19.54447373	-3.148967104	0.689011226	-4.570269667	4.87E-06	0.000724406
2	100038710	100039023	AluSq2:Alu:SINE	12.31146035	-2.661333226	0.751540409	-3.541171164	0.000398355	0.020122376
2	212496898	212497197	AluSq2:Alu:SINE	34.74816044	-1.931112125	0.583810593	-3.307771643	0.000940415	0.036214658
2	99839116	99839409	AluSq2:Alu:SINE	9.386133874	-3.07624802	0.819143594	-3.755444153	0.000173034	0.011006182
2	99993792	99994105	AluSq2:Alu:SINE	17.84694133	-2.973310833	0.756873768	-3.928410466	8.55E-05	0.006513505
2	102176983	102177271	AluSx:Alu:SINE	78.51116338	2.580937921	0.70622715	3.654543615	0.00025764	0.014342871
2	99549302	99549592	AluSx:Alu:SINE	122.9012594	-1.691669503	0.437664522	-3.865219636	0.000110989	0.00801416
2	99724267	99724572	AluSx:Alu:SINE	8.669803563	-2.578960151	0.802118706	-3.215185148	0.001303604	0.045056061

2	100057042	100057344	AluSx1:Alu:SINE	22.89280246	-2.288480989	0.660966595	-3.46232473	0.000535531	0.024889678
2	100058970	100059279	AluSx1:Alu:SINE	22.32028871	-2.425867417	0.654541468	-3.706208905	0.000210385	0.012632372
2	154857598	154857936	AluSx1:Alu:SINE	20.71391176	-2.912871402	0.676338936	-4.30682199	1.66E-05	0.001867284
2	99931626	99931915	AluSx1:Alu:SINE	17.2373349	-2.993087236	0.675828584	-4.428766861	9.48E-06	0.001259518
2	99958209	99958500	AluSz:Alu:SINE	11.26171641	-3.529651909	0.796567401	-4.431077529	9.38E-06	0.00125142
2	99991620	99991927	AluSz:Alu:SINE	17.991769	-3.219260842	0.761950223	-4.225027754	2.39E-05	0.002463413
2	100010993	100011286	AluY:Alu:SINE	16.2113818	-2.967672082	0.726316002	-4.085924136	4.39E-05	0.003962701
2	100078939	100079245	AluY:Alu:SINE	26.21954974	-3.405231588	0.682780181	-4.987302919	6.12E-07	0.000135618
2	131545573	131545872	AluY:Alu:SINE	15.72574102	-2.386874127	0.671237039	-3.555933284	0.00037664	0.019252582
2	131624093	131624426	AluY:Alu:SINE	9.867620063	-3.014104655	0.781016994	-3.859204958	0.000113757	0.008148462
2	167999451	167999761	AluY:Alu:SINE	10.24851283	-2.97669651	0.786772067	-3.783429326	0.000154682	0.010191734
2	212446989	212447296	AluY:Alu:SINE	35.47969879	-2.153003258	0.623512541	-3.453023183	0.000554341	0.025572578
2	212483746	212484035	AluY:Alu:SINE	34.46140738	-2.319386491	0.631405775	-3.673369145	0.000239373	0.013692064
2	212513530	212513839	AluY:Alu:SINE	50.0291863	-1.917823328	0.585888813	-3.273357135	0.001062781	0.039095069
2	99871925	99872232	AluY:Alu:SINE	9.14428218	-2.787318453	0.798565915	-3.49040499	0.000482289	0.023280324
2	99873892	99874191	AluY:Alu:SINE	11.17244765	-2.582555812	0.730976766	-3.533020381	0.000410841	0.020595456
2	99891828	99892121	AluY:Alu:SINE	15.12010259	-3.1645275	0.750808304	-4.214827519	2.50E-05	0.002532402
2	99985918	99986201	AluY:Alu:SINE	10.36905226	-2.749358263	0.809419764	-3.396702655	0.00068203	0.029259715
2	99995379	99995660	AluY:Alu:SINE	20.61641411	-3.471908908	0.742175887	-4.678013622	2.90E-06	0.000476137
2	212206586	212206887	AluYk4:Alu:SINE	11.37882206	-2.523929941	0.764185356	-3.302771928	0.000957342	0.036595781
2	212440548	212446198	HERVL-int:ERVL:LT	185.6685173	-1.866890594	0.535056512	-3.489146573	0.000484565	0.023354107
2	142405101	142405736	L1P3:L1:LINE	9.500402811	4.005879058	0.833195954	4.807847468	1.53E-06	0.000288771
2	99773691	99775704	L1PA11:L1:LINE	14.72913535	-2.856598744	0.74664918	-3.825891491	0.0001303	0.008924099
2	131628202	131629531	L1PA12:L1:LINE	148.7437766	-2.838531979	0.481380995	-5.896643218	3.71E-09	1.68E-06
2	131630168	131630732	L1PA13:L1:LINE	55.45053414	-2.691720283	0.503871909	-5.342072529	9.19E-08	2.59E-05
2	131632613	131633124	L1PA13:L1:LINE	14.48839153	-2.750206148	0.690281158	-3.984182553	6.77E-05	0.005507132
2	99626574	99628545	L1PA13:L1:LINE	17.11044305	-2.918662228	0.690862302	-4.22466564	2.39E-05	0.002463413
2	211888536	211894571	L1PA2:L1:LINE	126.6825686	-1.762947711	0.481248821	-3.663276943	0.000249009	0.014037549
2	94496017	94499162	L1PA2:L1:LINE	96.72978443	-1.447273145	0.395922401	-3.655446478	0.000256735	0.014318006
2	131613928	131617597	L1PA3:L1:LINE	158.1340269	-3.295579056	0.422213404	-7.80548184	5.93E-15	9.26E-12
2	137309506	137315534	L1PA3:L1:LINE	8.27108607	2.582096873	0.786561566	3.282765121	0.001027943	0.038264219
2	99739814	99741137	L1PA3:L1:LINE	38.40467986	-3.034074933	0.640778613	-4.734981584	2.19E-06	0.000395487
2	99954339	99954925	L1PA3:L1:LINE	38.97875601	-3.287193372	0.618057817	-5.318585543	1.05E-07	2.79E-05
2	131631502	131632587	L1PA4:L1:LINE	30.70256688	-2.359346586	0.601896818	-3.919852235	8.86E-05	0.006680244

2	132528881	132535004	L1PA4:L1:LINE	41.80733634	-2.374457441	0.699768136	-3.393206003	0.000690797	0.029302027
2	142430238	142432791	L1PA4:L1:LINE	22.01313091	3.49905705	0.796948953	4.39056609	1.13E-05	0.00142373
2	99908220	99909403	L1PA4:L1:LINE	83.96355148	-3.633800419	0.544476877	-6.673929733	2.49E-11	1.65E-08
2	11844304	11846272	L1PA5:L1:LINE	11.74538532	-2.991980427	0.826275088	-3.621046393	0.000293414	0.015637558
2	154767186	154769784	L1PA5:L1:LINE	23.35848264	-2.608093025	0.77680479	-3.357462592	0.000786614	0.031739971
2	234775486	234781643	L1PA5:L1:LINE	8.42012361	-2.587274332	0.813322467	-3.181117499	0.001467081	0.048889838
2	154838950	154845084	L1PA6:L1:LINE	19.13850544	-2.562529957	0.793908283	-3.227740547	0.001247721	0.043699051
2	168100710	168101366	L1PA7:L1:LINE	25.50049373	-2.897207372	0.618301589	-4.685751137	2.79E-06	0.000463373
2	212234004	212237135	L1PA7:L1:LINE	287.9686235	-1.993691691	0.50449325	-3.951869904	7.75E-05	0.006100106
2	212237134	212238540	L1PA7:L1:LINE	69.34504875	-1.895983654	0.55625632	-3.408471211	0.00065328	0.028415851
2	212274815	212275555	L1PA7:L1:LINE	77.84186976	-1.760037886	0.511471627	-3.441125166	0.0005793	0.026120762
2	99829200	99830299	L1PA8:L1:LINE	45.05116581	-3.596462716	0.579891773	-6.201955063	5.58E-10	3.06E-07
2	99933334	99933819	L1PA8:L1:LINE	18.6711757	-3.114345533	0.740356464	-4.206548716	2.59E-05	0.002612324
2	101098390	101102007	L1PA8A:L1:LINE	23.93197312	-2.730161862	0.6654552	-4.102698213	4.08E-05	0.003740024
2	100025210	100026690	L1PB1:L1:LINE	29.30702687	-2.798628482	0.601142802	-4.655513588	3.23E-06	0.00052026
2	154857401	154857585	L2a:L2:LINE	16.20774257	-2.321124716	0.675070385	-3.438344751	0.000585282	0.026300638
2	99940301	99940854	MER41A:ERV1:LTR	27.08296797	-2.654733242	0.641431785	-4.138761605	3.49E-05	0.003302948
2	131630732	131631257	MER4-int:ERV1:LT	19.08693138	-2.204646526	0.675382706	-3.264292238	0.001097379	0.039897843
2	26780450	26780632	MIRb:MIR:SINE	110.5711288	1.20320392	0.376063577	3.199469432	0.001376808	0.046285346
2	212440122	212440548	MLT2A1:ERVL:LTR	52.13975943	-2.352500904	0.600030615	-3.92063479	8.83E-05	0.006678445
2	212459913	212460266	THE1B:ERVL-MaLR	50.05929098	-2.313170189	0.510233277	-4.53355415	5.80E-06	0.000845019
2	99927822	99928194	THE1B:ERVL-MaLR	13.1619006	-3.286515295	0.758723904	-4.331635364	1.48E-05	0.001705673
2	168080643	168081664	THE1B-int:ERVL-M	13.1184186	-2.24074947	0.703852297	-3.183550695	0.001454806	0.048541728
2	212460266	212461716	THE1B-int:ERVL-M	161.414502	-2.216071631	0.518236198	-4.276180704	1.90E-05	0.002090794
2	212466712	212468298	THE1B-int:ERVL-M	198.172084	-1.853782934	0.505641939	-3.666196945	0.000246184	0.013928593
2	212505908	212508318	Tigger1:TcMar-Tig	26.54065021	-1.941738031	0.604092959	-3.214303367	0.001307614	0.045124979
3	112603244	112603521	AluSx1:Alu:SINE	16.54200674	2.483332756	0.7743086	3.207161533	0.001340517	0.045650742
3	23266649	23269368	L1P1:L1:LINE	59.42767406	-2.314990071	0.603966585	-3.832977071	0.000126602	0.008825669
3	141757129	141763153	L1PA2:L1:LINE	30.93556223	2.217229305	0.547624705	4.048811689	5.15E-05	0.004503418
3	53365276	53371325	L1PA2:L1:LINE	64.92743036	2.010388017	0.520158809	3.864950439	0.000111112	0.00801416
3	100509607	100515641	L1PA3:L1:LINE	47.52863541	-2.231842053	0.584191232	-3.820396355	0.000133237	0.009085452
3	189017229	189025537	L1PA3:L1:LINE	69.76882745	2.462442865	0.452137439	5.446226415	5.14E-08	1.61E-05
3	23349243	23355385	L1PA3:L1:LINE	198.2402297	-2.190832986	0.60256096	-3.635869453	0.000277045	0.015112278
3	125849810	125855962	L1PA4:L1:LINE	33.29387085	-2.236420555	0.600145697	-3.726462698	0.000194186	0.011874896

3	23296317	23301810	L1PA4:L1:LINE	83.39515361	-1.917282719	0.503295416	-3.80945794	0.000139272	0.009414713
3	55831626	55837762	L1PA4:L1:LINE	8.229961365	-2.771023099	0.831803725	-3.331342497	0.000864282	0.034038322
3	56213069	56219149	L1PA4:L1:LINE	8.700205724	-2.738887359	0.833557955	-3.285779164	0.001017007	0.038038501
3	15480181	15484403	L1PA5:L1:LINE	22.38442707	-2.061507719	0.643192553	-3.205117517	0.001350073	0.04578083
3	187052299	187058423	L1PA6:L1:LINE	72.8015667	-2.97273917	0.583507085	-5.094606816	3.49E-07	8.36E-05
3	23347104	23347712	L1PA6:L1:LINE	8.31982778	-3.024499555	0.820377771	-3.686715636	0.000227167	0.013138243
4	140729574	140729845	AluSc:Alu:SINE	27.02585621	-2.485797274	0.721003488	-3.447691052	0.0005654	0.025779056
4	140664826	140665128	AluSg:Alu:SINE	26.13362751	-2.058266459	0.604308373	-3.405986993	0.000659253	0.028635795
4	39502913	39503213	AluSg:Alu:SINE	13.81362238	-2.920275116	0.775993269	-3.763273771	0.000167703	0.010776844
4	140682882	140683177	AluSp:Alu:SINE	7.403312641	-2.813657171	0.828624003	-3.395577681	0.000684839	0.029259715
4	140624780	140625065	AluSq2:Alu:SINE	15.75906709	-2.204389842	0.685959643	-3.213585325	0.001310888	0.045188031
4	89239666	89239961	AluSx:Alu:SINE	12.99565091	-3.219714566	0.77934838	-4.13129051	3.61E-05	0.003383193
4	140712461	140712748	AluSx3:Alu:SINE	11.48371901	-2.528742035	0.791457618	-3.195044154	0.001398094	0.046950418
4	140725985	140726301	AluSz:Alu:SINE	13.65153606	-2.723327686	0.796576227	-3.418791065	0.000629	0.027551614
4	139046568	139046874	AluY:Alu:SINE	46.90234018	-1.838431796	0.512060789	-3.590260837	0.000330347	0.01742749
4	140660910	140661213	AluY:Alu:SINE	32.61093986	-1.813834256	0.564931935	-3.210712909	0.001324061	0.045449684
4	174984912	174989048	HERVK9-int:ERVK:	8.845753404	2.858686809	0.83655509	3.417212857	0.000632658	0.027634322
4	174979694	174982832	HERVL-int:ERVL:LT	9.720190341	3.685757008	0.796805376	4.625667845	3.73E-06	0.000580171
4	74449070	74449168	L1MEf:L1:LINE	32.0076003	2.263077902	0.712045218	3.17827835	0.001481524	0.049118355
4	86251145	86254837	L1PA2:L1:LINE	8.117942138	-2.724838852	0.833178423	-3.270414566	0.0010739	0.039318831
4	95171232	95177256	L1PA3:L1:LINE	147.1141744	-1.583059167	0.42427877	-3.731176947	0.000190587	0.011786598
4	95480626	95483004	L1PA3:L1:LINE	32.08859736	-3.046054889	0.822645688	-3.702754339	0.000213271	0.012667234
4	4013498	4019703	L1PA4:L1:LINE	13.94298505	-2.536074046	0.713713203	-3.553351731	0.000380356	0.01939125
4	9080578	9086679	L1PA4:L1:LINE	68.54598228	-1.55905715	0.451810599	-3.450687418	0.000559161	0.025605792
4	95133878	95140064	L1PA5:L1:LINE	157.5772485	-2.004470373	0.458413807	-4.372622168	1.23E-05	0.001509485
4	38824163	38824458	L2a:L2:LINE	11.41212366	-3.503963241	0.79872719	-4.386933718	1.15E-05	0.001441891
4	75047494	75047704	MER20:hAT-Charli	9.432317781	-2.705565746	0.79596026	-3.39912164	0.000676026	0.029201911
4	174983875	174984522	SVA_A:SVA:Retrop	8.062451314	3.239702159	0.826579961	3.919405639	8.88E-05	0.006680244
4	150963149	150964838	SVA_B:SVA:Retrop	78.13113424	-2.3413167	0.720802642	-3.248207712	0.001161345	0.041689601
4	98366453	98368013	THE1B-int:ERVL-M	34.32749732	-2.020713757	0.577057093	-3.50175707	0.000462201	0.022591795
5	70110690	70110972	AluJb:Alu:SINE	7.397762784	-2.870079912	0.826040496	-3.474502675	0.000511801	0.024072006
5	69652415	69652519	AluSq2:Alu:SINE	9.587275347	-3.129008925	0.81682673	-3.830688702	0.000127785	0.008842895
5	95794820	95795118	AluSz:Alu:SINE	67.12058965	1.508223359	0.445316025	3.38686073	0.000706973	0.029491397
5	132822351	132822661	AluY:Alu:SINE	118.331848	-2.720379111	0.763308197	-3.563932789	0.00036534	0.018890595

5	15813202	15814587	L1PA7:L1:LINE	16.15191985	-2.384358579	0.669640074	-3.560656945	0.000369928	0.01903332
5	15870695	15872406	L1PA7:L1:LINE	21.38828924	-3.209846051	0.743723415	-4.315913668	1.59E-05	0.001798545
5	15872433	15876411	L1PA7:L1:LINE	33.93529087	-3.062889998	0.601461142	-5.092415425	3.54E-07	8.36E-05
5	151661901	151662129	L2a:L2:LINE	71.65109192	2.435794584	0.743932564	3.274214225	0.001059563	0.039022638
5	139475741	139475954	LTR33:ERVL:LTR	81.84887548	1.300101551	0.384545346	3.380879696	0.000722542	0.03000758
5	151661681	151661886	MER3:hAT-Charlie	65.73974018	2.348205398	0.730245933	3.215636394	0.001301556	0.045056061
5	43665240	43666629	SVA_B:SVA:Retrop	12.76006212	-3.220336559	0.816436642	-3.944380242	8.00E-05	0.006169597
5	70528190	70528393	THE1B:ERVL-MaLR	14.0350303	-2.787281124	0.764395005	-3.646388456	0.000265952	0.014681215
5	15743104	15744696	THE1C-int:ERVL-M	18.24724328	-2.876264387	0.721447657	-3.98679566	6.70E-05	0.005489742
6	87086803	87087087	AluSx1:Alu:SINE	14.26700558	-2.879768039	0.716710265	-4.018036549	5.87E-05	0.004980419
6	30806190	30806502	AluSz:Alu:SINE	21.73769007	-3.767946927	0.703140217	-5.35874188	8.38E-08	2.40E-05
6	31869703	31869997	AluY:Alu:SINE	14.09074681	-2.306089062	0.707488144	-3.259544461	0.001115913	0.040386189
6	151867443	151867765	L1P3:L1:LINE	24.12892487	-2.344954423	0.63567117	-3.688942547	0.000225188	0.013096552
6	151931455	151933731	L1PA2:L1:LINE	64.74577009	-2.239079534	0.517938498	-4.323060639	1.54E-05	0.001765193
6	151863059	151863690	L1PA5:L1:LINE	56.21315912	-1.874129699	0.577504824	-3.245219124	0.001173603	0.041984866
6	115935045	115941505	L1PA7:L1:LINE	86.03840722	1.863500525	0.493170488	3.778613216	0.000157704	0.010303888
6	151866336	151866866	L1PA7:L1:LINE	50.19872659	-2.367512628	0.584025229	-4.053784852	5.04E-05	0.004446056
6	151967187	151968062	L1PA7:L1:LINE	88.21015852	-1.804766719	0.469991742	-3.839996663	0.000123036	0.008673898
6	151968061	151968548	L1PA7:L1:LINE	31.92917846	-2.356681372	0.567241267	-4.154636672	3.26E-05	0.003140497
6	71355361	71361788	L1PA7:L1:LINE	19.56166275	-2.419071998	0.728701582	-3.319701863	0.000901136	0.035004213
6	165764432	165765849	LTR12C:ERV1:LTR	13.83326113	-2.703916367	0.837380973	-3.229015771	0.00124217	0.04359241
6	30806717	30808005	LTR12C:ERV1:LTR	129.61572	-3.366254566	0.435856213	-7.723314398	1.13E-14	1.54E-11
6	165780893	165781351	MSTB2:ERVL-MaLR	7.589596983	-2.767007426	0.830834424	-3.33039574	0.000867226	0.034111267
6	151898400	151900119	SVA_D:SVA:Retrop	473.252307	-2.521634192	0.533434388	-4.727168414	2.28E-06	0.000404002
6	53304468	53306203	SVA_D:SVA:Retrop	76.20455454	-1.884718099	0.574828261	-3.278749891	0.00104268	0.03860703
7	17345034	17345326	AluSc:Alu:SINE	117.4200707	-2.281784149	0.538608629	-4.236441871	2.27E-05	0.002396026
7	17310536	17310835	AluSq2:Alu:SINE	25.14512779	-3.372733103	0.649580408	-5.192171842	2.08E-07	5.36E-05
7	17344621	17344927	AluSx:Alu:SINE	144.8851432	-2.41402574	0.547912352	-4.405861144	1.05E-05	0.001359767
7	17324520	17324815	AluSx3:Alu:SINE	17.86729731	-2.669999077	0.662279563	-4.03152872	5.54E-05	0.004780864
7	8461548	8461858	AluYb8:Alu:SINE	9.4706935	-2.818643791	0.798016954	-3.532060036	0.000412336	0.020637271
7	64990915	64999343	HERV3-int:ERV1:LT	267.4350388	1.377353752	0.314690772	4.376848239	1.20E-05	0.001492244
7	8672103	8672480	L1PA3:L1:LINE	7.346004207	-2.755446349	0.831025973	-3.315716281	0.000914086	0.035331452
7	8726340	8732368	L1PA3:L1:LINE	63.40153892	-3.25925388	0.510020537	-6.390436554	1.65E-10	9.93E-08
7	65480283	65486433	L1PA4:L1:LINE	178.0582268	2.898501345	0.552683759	5.24441201	1.57E-07	4.11E-05

7	8496881	8497980	L1PA5:L1:LINE	98.91197211	-3.480512998	0.466546403	-7.460164679	8.64E-14	1.04E-10
7	8732890	8734564	L1PA7:L1:LINE	12.82826747	-3.01509641	0.733679971	-4.109552572	3.96E-05	0.00364141
7	8734830	8739610	L1PA7:L1:LINE	21.98425616	-4.008450655	0.720741639	-5.561563862	2.67E-08	8.91E-06
7	73749695	73750685	LTR5B:ERVK:LTR	39.36939904	-2.095335562	0.578239131	-3.623648851	0.000290476	0.01556064
7	131503890	131504051	MIR3:MIR:SINE	14.93608663	2.786532284	0.820994744	3.394092722	0.000688564	0.029302027
7	8524931	8526510	THE1A-int:ERVL-M	78.8030321	-2.440823613	0.554473503	-4.402056367	1.07E-05	0.001378144
8	128015494	128015790	AluSc:Alu:SINE	39.76038166	-2.569142923	0.644031874	-3.989154923	6.63E-05	0.005449736
8	127950898	127951191	AluSg:Alu:SINE	18.3613705	-2.293554497	0.685454216	-3.346036019	0.000819757	0.032655415
8	133521310	133521597	AluSg4:Alu:SINE	26.75049443	-1.861035352	0.575639869	-3.232985505	0.001225038	0.043279587
8	127804927	127805229	AluSp:Alu:SINE	116.7582309	-1.51489764	0.450548111	-3.362343783	0.000772838	0.031392298
8	127934971	127935252	AluSp:Alu:SINE	23.0821831	-1.994739759	0.608329225	-3.279046405	0.001041585	0.03860703
8	127925635	127925938	AluSq:Alu:SINE	21.28200273	-2.03198308	0.632020357	-3.215059542	0.001304175	0.045056061
8	127816510	127816783	AluSq2:Alu:SINE	22.81071578	-1.81580662	0.571524514	-3.177128148	0.001487413	0.049209104
8	128042790	128043063	AluSx:Alu:SINE	39.03668157	-2.111328214	0.58216805	-3.626664523	0.000287106	0.015486364
8	132674608	132674905	AluSx:Alu:SINE	18.17249007	2.879484406	0.678715332	4.242550993	2.21E-05	0.002360711
8	127808747	127809059	AluSx1:Alu:SINE	21.42932521	-2.568469056	0.770949405	-3.331566299	0.000863587	0.034038322
8	133497456	133497771	AluSx1:Alu:SINE	30.21166423	-2.332792322	0.631103578	-3.696369981	0.000218704	0.012790918
8	133538232	133538541	AluSx1:Alu:SINE	53.14934287	-1.898109848	0.468785597	-4.048993528	5.14E-05	0.004503418
8	127841263	127841552	AluSz:Alu:SINE	28.85593705	-1.999460011	0.607067988	-3.293634403	0.00098901	0.037494645
8	127983876	127984176	AluSz:Alu:SINE	82.68020845	-1.557196838	0.474074249	-3.284710866	0.001020871	0.038091774
8	128060905	128061208	AluSz:Alu:SINE	24.09739389	-2.257070095	0.672779617	-3.354843159	0.0007941	0.031918321
8	127992104	127992388	AluSz6:Alu:SINE	26.13992628	-2.230567991	0.595904636	-3.743162676	0.000181718	0.011396085
8	128015105	128015386	AluSz6:Alu:SINE	22.80241125	-2.188288179	0.653023352	-3.351010609	0.000805172	0.032197609
8	127821529	127821830	AluY:Alu:SINE	50.90755642	-2.046553696	0.627547606	-3.261192737	0.001109446	0.040279758
8	127844703	127845012	AluY:Alu:SINE	55.74493112	-1.938668119	0.599385155	-3.234427986	0.001218867	0.04321853
8	127924501	127924810	AluY:Alu:SINE	40.63453373	-1.963463912	0.589867985	-3.328649736	0.000872681	0.034282634
8	127929516	127929782	AluY:Alu:SINE	23.49727135	-2.396515717	0.732640893	-3.27106464	0.001071434	0.039310706
8	127952770	127953063	AluY:Alu:SINE	42.04203305	-2.067676212	0.587420013	-3.519928103	0.000431664	0.02150449
8	128021290	128021592	AluY:Alu:SINE	83.88569388	-1.886952158	0.572283869	-3.297231076	0.000976431	0.037188935
8	103602996	103609018	L1PA2:L1:LINE	44.87095122	-2.235040291	0.639311179	-3.496013153	0.000472265	0.022957091
8	103783269	103789216	L1PA2:L1:LINE	25.02680944	-3.221388559	0.696585269	-4.624543044	3.75E-06	0.000580441
8	103751167	103756353	L1PA4:L1:LINE	15.76465339	-2.952624345	0.741004143	-3.984625963	6.76E-05	0.005507132
8	103549090	103553602	L1PA5:L1:LINE	114.5497593	-2.204498317	0.608058172	-3.625472725	0.000288433	0.015531141
8	103738312	103739541	L1PA5:L1:LINE	16.82727945	-2.544132394	0.730400235	-3.483203142	0.000495452	0.023646178

8	103790883	103795584	L1PA5:L1:LINE	55.70069258	-2.910162741	0.605030513	-4.809943762	1.51E-06	0.000288771
8	140382163	140388390	L1PA5:L1:LINE	51.32639405	-2.25730648	0.605541193	-3.727750494	0.000193197	0.011874896
8	103951313	103957769	L1PA7:L1:LINE	23.37729314	-2.488927473	0.671783066	-3.704957148	0.000211426	0.012632372
8	39179932	39181535	LTR12C:ERV1:LTR	23.38025593	-2.457386627	0.756122243	-3.249985899	0.001154107	0.041477475
8	128100639	128101309	LTR8A:ERV1:LTR	184.674047	-1.285971541	0.34814587	-3.693772215	0.000220952	0.012874149
8	130393752	130394816	MER11B:ERVK:LTR	11.35434914	-2.496355074	0.77001882	-3.241940338	0.001187189	0.042277185
8	127939637	127939772	MIRb:MIR:SINE	41.11131823	-1.905442277	0.540674558	-3.524194453	0.000424772	0.021225707
8	127947602	127947679	MIRb:MIR:SINE	34.92712695	-2.187265529	0.561502861	-3.895377353	9.80E-05	0.007238945
8	123209795	123210136	MLT1I:ERVL-MaLR	39.45826455	1.68752658	0.524320848	3.21849987	0.00128863	0.044816495
8	103562119	103563698	THE1B-int:ERVL-M	18.42307376	-2.422871677	0.684506655	-3.539588195	0.000400752	0.020178958
9	63818917	63819225	AluSq:Alu:SINE	62.52881299	-1.74674625	0.514874966	-3.392563954	0.000692418	0.029302027
9	86157088	86163107	L1PA2:L1:LINE	35.42081689	1.901636159	0.568659084	3.344070661	0.000825587	0.032762274
9	41149958	41155994	L1PA3:L1:LINE	42.88441508	-1.728829645	0.531472788	-3.252903412	0.001142323	0.041101262
9	68275326	68281363	L1PA3:L1:LINE	45.03853583	-1.650039588	0.517836589	-3.186409811	0.001440504	0.048115912
9	65291009	65292463	L1PB1:L1:LINE	19.00634972	-2.33484756	0.708923314	-3.293512165	0.000989441	0.037494645
9	65292463	65295683	L1PB1:L1:LINE	16.45006266	-3.059810798	0.773398551	-3.956318245	7.61E-05	0.006017996
9	99865852	99866047	MIR3:MIR:SINE	24.45718069	-3.118113836	0.761850506	-4.092815862	4.26E-05	0.003869068
10	14582364	14582676	AluSc8:Alu:SINE	12.19115625	-2.79847792	0.833662128	-3.356849046	0.000788361	0.031769439
10	843416	843718	AluSq2:Alu:SINE	12.03073306	-2.402144226	0.711045374	-3.378327622	0.000729281	0.030127231
10	77820063	77820366	AluSx1:Alu:SINE	35.29957323	-1.771544046	0.515784381	-3.434660123	0.000593297	0.026508258
10	91749615	91755638	L1PA3:L1:LINE	73.81834889	1.612068877	0.491264733	3.281466728	0.001032687	0.03839506
10	11972382	11974769	L1PA5:L1:LINE	38.05986462	-2.131081502	0.503521901	-4.232351159	2.31E-05	0.002423661
10	21778609	21781440	L1PA5:L1:LINE	14.812017	-2.201644566	0.655496248	-3.358744726	0.000782974	0.031633956
10	92375117	92375955	SVA_F:SVA:Retrop	17.59914333	-3.020460392	0.810125233	-3.728387008	0.000192709	0.011870816
11	57994162	58000000	L1PA2:L1:LINE	15.93093797	2.37230033	0.658468418	3.602754917	0.000314862	0.016723583
11	75748616	75754649	L1PA2:L1:LINE	21.1988437	2.542042673	0.596440457	4.262022543	2.03E-05	0.002219976
11	73297944	73298234	L2b:L2:LINE	19.66967147	-2.370976618	0.72604793	-3.265592422	0.001092354	0.039877726
11	125089312	125090854	LTR12C:ERV1:LTR	46.13507898	-2.753562348	0.71756005	-3.837396394	0.000124346	0.00872683
11	73272218	73272327	MIRc:MIR:SINE	15.59809819	-2.552649179	0.728966849	-3.501735617	0.000462238	0.022591795
11	129128567	129129672	SVA_B:SVA:Retrop	11.63084638	-3.061384748	0.822770688	-3.720823787	0.000198574	0.012029751
11	62841623	62841809	U2:snRNA:snRNA	175.1824379	-2.319160974	0.714410295	-3.246259172	0.001169323	0.041879745
12	116146116	116146418	AluSc:Alu:SINE	18.08178402	-2.812273453	0.766901699	-3.667058579	0.000245357	0.013928593
12	116206074	116206325	AluSx:Alu:SINE	12.72425097	-2.791505728	0.767116953	-3.638957161	0.000273744	0.015025148
12	101185569	101185870	AluSx1:Alu:SINE	11.66381662	-2.70967242	0.785230861	-3.450797152	0.000558933	0.025605792

12	48860077	48860380	AluSz6:Alu:SINE	13.57008922	-2.817755857	0.805495096	-3.498166372	0.000468469	0.022824881
12	95749284	95752709	HERVH-int:ERV1:L	13.6094543	-2.579037054	0.780722252	-3.303398932	0.000955204	0.036559004
12	101152060	101155141	L1PA2:L1:LINE	55.42075839	-2.996559877	0.679524243	-4.409790976	1.03E-05	0.001345511
12	8301068	8307182	L1PA4:L1:LINE	64.44756617	-1.585414742	0.42382956	-3.740689397	0.000183516	0.01148576
12	95775205	95781318	L1PA5:L1:LINE	70.53531788	-2.318945239	0.6177383	-3.753928222	0.000174085	0.011028074
12	116660542	116661920	LTR12C:ERV1:LTR	21.70056664	-3.055325761	0.701431512	-4.35584331	1.33E-05	0.001601413
12	122591840	122593220	LTR12C:ERV1:LTR	77.26067498	-1.652397983	0.472057091	-3.500419787	0.000464526	0.022668144
12	119179222	119179304	MIR3:MIR:SINE	54.07432995	2.177655468	0.497388828	4.378175276	1.20E-05	0.001489098
12	52168981	52169353	MSTB:ERVL-MaLR	21.03809531	1.964786622	0.578944732	3.393737801	0.000689457	0.029302027
12	100293530	100295757	SVA_D:SVA:Retrop	53.07154223	-2.665329068	0.692580404	-3.848403812	0.00011889	0.008457978
12	48915110	48916626	SVA_D:SVA:Retrop	84.12122183	-1.821971693	0.5435293	-3.352113108	0.000801972	0.032151992
13	49158536	49163769	L1P2:L1:LINE	78.36712281	-1.995013936	0.556243173	-3.586585924	0.000335036	0.017556208
13	96204174	96208006	L1PA11:L1:LINE	20.8297162	-2.06588049	0.597861983	-3.455447161	0.000549381	0.025418835
13	49013626	49019628	L1PA3:L1:LINE	104.5800187	-2.041546328	0.491090924	-4.157165662	3.22E-05	0.003115561
13	43710923	43716379	L1PA4:L1:LINE	39.15986724	-2.660410907	0.719584854	-3.69714689	0.000218036	0.012775775
13	96326180	96332208	L1PA4:L1:LINE	46.07123257	-1.827566777	0.530511667	-3.444913452	0.000571242	0.025968657
13	49118181	49124497	L1PA7:L1:LINE	48.31476876	-1.786076554	0.545482712	-3.274304602	0.001059224	0.039022638
13	113621366	113622349	LTR13:ERVK:LTR	22.56105623	-2.161637518	0.650999637	-3.320489589	0.000898597	0.034992626
13	97358238	97358429	MIR3:MIR:SINE	19.41708991	-2.595336072	0.70082055	-3.70328192	0.000212828	0.012667234
14	65522585	65528687	L1PA5:L1:LINE	67.51322061	-2.137064242	0.573106227	-3.728914715	0.000192306	0.011869398
14	66701365	66707789	L1PA8A:L1:LINE	37.12158838	-2.000023987	0.589478202	-3.39287183	0.00069164	0.029302027
14	65632981	65634580	SVA_D:SVA:Retrop	182.8006542	-1.572652038	0.452109909	-3.478472837	0.00050428	0.023872272
14	34546713	34546877	U1:snRNA:snRNA	2792.545534	-2.4831681	0.6191897	-4.010351107	6.06E-05	0.005090025
14	34556225	34556389	U1:snRNA:snRNA	2540.895852	-2.671157037	0.624379978	-4.278095282	1.88E-05	0.002087597
15	89200800	89201095	AluSq:Alu:SINE	54.14279311	1.826053139	0.44207961	4.130597968	3.62E-05	0.003383242
15	61912917	61913284	L1PA8A:L1:LINE	7.698668043	-2.64441487	0.803210451	-3.292306353	0.000993693	0.03752603
15	89196302	89196472	MIR3:MIR:SINE	76.16713526	1.834650304	0.43625429	4.205460773	2.61E-05	0.002616483
15	89198595	89198679	MIR3:MIR:SINE	39.04401034	1.911649228	0.513012759	3.72631907	0.000194296	0.011874896
15	89200250	89200502	SVA_F:SVA:Retrop	131.0737153	2.091885195	0.442393442	4.728562853	2.26E-06	0.000403531
16	9110767	9111055	AluSp:Alu:SINE	44.76850245	1.628672823	0.470427814	3.462109962	0.000535958	0.024889678
16	71567127	71567437	AluSx:Alu:SINE	10.61955485	2.589543317	0.764063597	3.389172481	0.000701039	0.029425405
16	20310748	20311056	AluSx1:Alu:SINE	48.0182916	-3.097081537	0.660092051	-4.691893398	2.71E-06	0.000452078
16	8783193	8783543	L2c:L2:LINE	78.05722928	1.492882269	0.392433277	3.804168395	0.000142281	0.009556106
16	71588846	71590265	SVA_D:SVA:Retrop	8.985709468	2.939944893	0.781959623	3.75971445	0.000170107	0.010890681

17	42403073	42403228	AluJr:Alu:SINE	59.23288232	2.574460523	0.709837657	3.626830021	0.000286922	0.015486364
17	44019706	44020376	HERVL66-int:ERVL	22.32586283	-3.102738764	0.827169587	-3.751030999	0.000176109	0.01113372
17	44019061	44019706	LTR66:ERVL:LTR	10.31047111	-2.660468427	0.83734575	-3.177263905	0.001486717	0.049209104
17	32941260	32941516	MER102a:hAT-Cha	47.16195639	1.760999298	0.51496209	3.419667842	0.000626976	0.027508592
17	43239924	43240111	U2:snRNA:snRNA	7.57264295	-2.882391393	0.825795844	-3.490440662	0.000482225	0.023280324
17	43273104	43273292	U2:snRNA:snRNA	9.774369169	3.905657265	0.837750004	4.662079675	3.13E-06	0.000511845
18	35897480	35903637	L1PA4:L1:LINE	7.709415278	-2.681314191	0.834571689	-3.21280272	0.001314465	0.045211531
19	13834729	13834886	AluJr:Alu:SINE	36.20543399	-2.174224251	0.546066743	-3.981608984	6.85E-05	0.005538269
19	13835344	13835653	AluSx:Alu:SINE	45.93234369	-2.42354079	0.531029251	-4.56385554	5.02E-06	0.000743367
19	13834886	13835207	AluSx1:Alu:SINE	48.019372	-1.915617089	0.507988776	-3.770983102	0.000162606	0.010579871
19	13837775	13838087	AluSx1:Alu:SINE	61.34179426	-2.386778966	0.462315402	-5.162663744	2.43E-07	6.18E-05
19	44751589	44751889	AluSx1:Alu:SINE	13.34869886	-2.410112156	0.739527636	-3.258988628	0.001118101	0.040416004
19	20154965	20156337	HERVH-int:ERV1:L	10.43277326	-2.854366794	0.83074237	-3.435922972	0.000590539	0.026498734
19	15056138	15056368	L3:CR1:LINE	19.1785401	-2.692969688	0.830533891	-3.24245611	0.001185042	0.042248912
19	2715361	2715715	MLT1E1A:ERVL-M	22.09887453	2.326917848	0.675918664	3.442600377	0.00057615	0.02608631
19	11676262	11677933	SVA_B:SVA:Retrop	118.3803529	-2.557136018	0.633980131	-4.033463972	5.50E-05	0.004767986
20	58678370	58678657	AluSg:Alu:SINE	139.5574151	-1.461369652	0.390174933	-3.745421676	0.000180091	0.011316746
20	51634574	51634869	AluSp:Alu:SINE	12.94866653	-2.517042523	0.701649294	-3.587322819	0.000334091	0.017551188
20	63953098	63953408	AluSp:Alu:SINE	16.79128395	-2.265032704	0.666036638	-3.400762922	0.000671981	0.029067358
20	51632845	51633153	AluSx1:Alu:SINE	23.12617093	-3.278173011	0.666480922	-4.918629932	8.72E-07	0.000183909
20	62806260	62806496	AluSx1:Alu:SINE	40.89432561	-1.804377591	0.513327032	-3.515064427	0.000439647	0.021794643
20	14806154	14812579	L1PA7:L1:LINE	21.73196166	-2.135173698	0.582936771	-3.66278781	0.000249485	0.014039049
20	62366948	62367030	LTR35B:ERV1:LTR	43.8451593	-2.079841558	0.531022505	-3.916673093	8.98E-05	0.006723979
20	63659689	63660674	LTR5B:ERVK:LTR	91.33782717	-1.680973987	0.458319302	-3.667691887	0.00024475	0.013923102
20	63529149	63529330	MER31-int:ERV1:L	123.0404042	-1.360614756	0.35900537	-3.789956555	0.000150674	0.010005592
20	38129160	38129333	MIR3:MIR:SINE	170.9386726	2.102296032	0.620633318	3.387339949	0.000705739	0.029491397
21	8232170	8232399	AluSx1:Alu:SINE	25.14175561	4.500803512	0.722838817	6.226565878	4.77E-10	2.71E-07
21	8235599	8235801	AluSx1:Alu:SINE	7.948299849	3.587598948	0.824943901	4.348900508	1.37E-05	0.001605796
21	8414762	8414991	AluSx1:Alu:SINE	21.66382878	4.356759233	0.720803381	6.044310206	1.50E-09	7.32E-07
21	8418002	8418204	AluSx1:Alu:SINE	7.948299849	3.587598948	0.824943901	4.348900508	1.37E-05	0.001605796
21	8462565	8462767	AluSx1:Alu:SINE	7.948299849	3.587598948	0.824943901	4.348900508	1.37E-05	0.001605796
21	8414175	8414470	AluSz:Alu:SINE	10.97579738	4.104725228	0.803066472	5.111314405	3.20E-07	7.81E-05
21	8458737	8459032	AluSz:Alu:SINE	10.97579738	4.104725228	0.803066472	5.111314405	3.20E-07	7.81E-05
21	14326861	14332307	L1P3:L1:LINE	17.68367056	4.435237391	0.770167882	5.758792979	8.47E-09	3.44E-06

21	14933877	14939951	L1PA5:L1:LINE	138.9847068	-2.255996823	0.674715511	-3.34362674	0.000826909	0.032773095
21	8397806	8401980	LSU-rRNA_Hsa:rRI	863.1896273	-2.470417715	0.568152077	-4.348162779	1.37E-05	0.001605796
22	42557273	42557585	AluSp:Alu:SINE	16.86115214	-3.219224539	0.755940058	-4.258571171	2.06E-05	0.002238813
22	42564427	42564730	AluSq2:Alu:SINE	19.77992497	-2.919596341	0.709304168	-4.116141528	3.85E-05	0.003570415
22	42567747	42568029	AluSq2:Alu:SINE	21.4644437	-2.930775847	0.754336346	-3.885237483	0.00010223	0.007477148
22	42559799	42560114	AluSx:Alu:SINE	10.61615444	-2.624291699	0.808773833	-3.244778195	0.001175421	0.042001815
22	42571478	42571721	AluSx1:Alu:SINE	328.263528	-3.625303857	0.426190365	-8.506301767	1.80E-17	5.10E-14
22	42572751	42572969	AluSx1:Alu:SINE	35.32791595	-2.13884028	0.543280665	-3.936897477	8.25E-05	0.006302847
22	42568690	42568922	AluSz:Alu:SINE	12.17674532	-2.713347932	0.807429006	-3.360478646	0.000778075	0.031517606
22	42573243	42573559	AluY:Alu:SINE	88.86252843	-2.288026061	0.5468844	-4.183747169	2.87E-05	0.002842943
22	42567382	42567700	AluYb8:Alu:SINE	19.49142013	-2.59885685	0.790164668	-3.289006653	0.001005416	0.037828944
22	42571722	42572117	L1MB7:L1:LINE	197.124559	-3.344640637	0.468561408	-7.138105234	9.46E-13	8.96E-10
22	42502097	42502196	MIR3:MIR:SINE	29.41014159	-2.738561363	0.628891314	-4.354586082	1.33E-05	0.001601413
22	42555410	42555509	MIR3:MIR:SINE	29.41014159	-2.738561363	0.628891314	-4.354586082	1.33E-05	0.001601413
22	42570794	42570947	MLT1J1:ERVL-MaL	81.22358269	-2.568220926	0.584233076	-4.395884166	1.10E-05	0.001400606
22	40501391	40501853	SVA_F:SVA:Retrop	18.621571	-2.975499386	0.792174166	-3.756117675	0.00017257	0.010999601
X	146145594	146145903	AluSz:Alu:SINE	13.85748992	-2.460812233	0.767430122	-3.206561957	0.001343314	0.045650742
X	25882278	25885802	L1P2:L1:LINE	18.11778049	-2.113842478	0.643287523	-3.285999501	0.001016212	0.038038501
X	104826133	104829583	L1P3:L1:LINE	8.717254028	-2.454680029	0.771880888	-3.180128006	0.0014721	0.04896183
X	104672099	104678122	L1PA2:L1:LINE	28.83214905	-4.376787167	0.766645126	-5.709013229	1.14E-08	4.38E-06
X	104904301	104910378	L1PA2:L1:LINE	20.3590916	-3.973731554	0.783008806	-5.074951296	3.88E-07	9.03E-05
X	105041515	105043241	L1PA3:L1:LINE	11.11015817	-3.293246161	0.810382866	-4.063815141	4.83E-05	0.004283355
X	85320258	85323630	L1PA3:L1:LINE	54.36324137	-1.913881797	0.591605816	-3.235062513	0.001216161	0.04321038
X	105029253	105035391	L1PA4:L1:LINE	10.84547984	-3.292398146	0.809989646	-4.064741026	4.81E-05	0.004278542
X	105073045	105079149	L1PA4:L1:LINE	8.41918773	-2.890244045	0.826777433	-3.49579455	0.000472652	0.022957091
X	129240861	129246997	L1PA4:L1:LINE	13.77373636	-2.649449045	0.703435625	-3.766441379	0.000165591	0.010707186
X	25772423	25775961	L1PA4:L1:LINE	9.502068122	-3.024454923	0.82194505	-3.679631534	0.000233571	0.013434
X	104846199	104848241	L1PA5:L1:LINE	8.56010707	-2.95254377	0.824050774	-3.582963407	0.000339718	0.01771242
X	104889804	104895934	L1PA5:L1:LINE	17.02765458	-4.067569022	0.771604402	-5.271573118	1.35E-07	3.58E-05
X	105684062	105690225	L1PA6:L1:LINE	8.322856514	-2.865048707	0.827591155	-3.461913154	0.00053635	0.024889678
X	104602037	104608348	L1PA7:L1:LINE	10.75059263	-2.686008892	0.836597669	-3.210633966	0.001324425	0.045449684
X	129419635	129423210	L1PA7:L1:LINE	23.43093723	-2.379949763	0.705256715	-3.374586463	0.000739267	0.030379005
X	94481142	94483200	L1PA8A:L1:LINE	11.65685318	-3.500431611	0.799381567	-4.3789246	1.19E-05	0.001489098
Y	24776006	24782046	L1PA2:L1:LINE	8.183513625	-2.69393479	0.834642117	-3.227652588	0.001248105	0.043699051

Supplemental Table S5. Location of differentially expressed mobile elements that overlap differentially expressed genes.

Chr	Start	End
chr1	109203930	109204222
chr1	109206011	109206314
chr1	109203594	109203905
chr1	108578637	108579660
chr1	197508889	197509568
chr1	108576306	108578640
chr1	109176604	109177644
chr2	99548467	99548746
chr2	212372467	212372778
chr2	212514524	212514805
chr2	100001412	100001726
chr2	99700123	99700418
chr2	100082798	100083115
chr2	99824120	99824414
chr2	99929110	99929357
chr2	100062968	100063273
chr2	100038710	100039023
chr2	212496898	212497197
chr2	99839116	99839409
chr2	99993792	99994105
chr2	102176983	102177271
chr2	99549302	99549592
chr2	99724267	99724572
chr2	100057042	100057344
chr2	100058970	100059279
chr2	154857598	154857936
chr2	99931626	99931915
chr2	99958209	99958500
chr2	99991620	99991927
chr2	100010993	100011286
chr2	100078939	100079245
chr2	131624093	131624426
chr2	167999451	167999761
chr2	212446989	212447296
chr2	212483746	212484035
chr2	212513530	212513839
chr2	99871925	99872232
chr2	99873892	99874191
chr2	99891828	99892121
chr2	99985918	99986201
chr2	99995379	99995660
chr2	212206586	212206887
chr2	212440548	212446198
chr2	99773691	99775704

chr2	99626574	99628545
chr2	211888536	211894571
chr2	131613928	131617597
chr2	99739814	99741137
chr2	99954339	99954925
chr2	99908220	99909403
chr2	154767186	154769784
chr2	154838950	154845084
chr2	168100710	168101366
chr2	212234004	212237135
chr2	212237134	212238540
chr2	212274815	212275555
chr2	99829200	99830299
chr2	99933334	99933819
chr2	100025210	100026690
chr2	154857401	154857585
chr2	99940301	99940854
chr2	26780450	26780632
chr2	212440122	212440548
chr2	212459913	212460266
chr2	99927822	99928194
chr2	168080643	168081664
chr2	212460266	212461716
chr2	212466712	212468298
chr2	212505908	212508318
chr3	23266649	23269368
chr3	23349243	23355385
chr3	23296317	23301810
chr3	55831626	55837762
chr3	56213069	56219149
chr3	187052299	187058423
chr3	23347104	23347712
chr4	89239666	89239961
chr4	95171232	95177256
chr4	95480626	95483004
chr4	38824163	38824458
chr4	75047494	75047704
chr5	95794820	95795118
chr5	15813202	15814587
chr5	15870695	15872406
chr5	15872433	15876411
chr5	151661901	151662129
chr5	151661681	151661886
chr5	15743104	15744696
chr6	87086803	87087087
chr6	115935045	115941505
chr7	17345034	17345326

chr7	17310536	17310835
chr7	17344621	17344927
chr7	17324520	17324815
chr7	8461548	8461858
chr7	8672103	8672480
chr7	8726340	8732368
chr7	8496881	8497980
chr7	8732890	8734564
chr7	8734830	8739610
chr7	131503890	131504051
chr7	8524931	8526510
chr8	132674608	132674905
chr8	103602996	103609018
chr8	103783269	103789216
chr8	103751167	103756353
chr8	103549090	103553602
chr8	103738312	103739541
chr8	103790883	103795584
chr8	103951313	103957769
chr8	127947602	127947679
chr8	123209795	123210136
chr8	103562119	103563698
chr9	63818917	63819225
chr9	41149958	41155994
chr9	99865852	99866047
chr11	73297944	73298234
chr11	125089312	125090854
chr11	73272218	73272327
chr12	101185569	101185870
chr12	119179222	119179304
chr12	52168981	52169353
chr13	97358238	97358429
chr15	89200800	89201095
chr15	89196302	89196472
chr15	89198595	89198679
chr15	89200250	89200502
chr16	20310748	20311056
chr16	8783193	8783543
chr17	42403073	42403228
chr17	44019706	44020376
chr17	44019061	44019706
chr19	13834729	13834886
chr19	13835344	13835653
chr19	13834886	13835207
chr19	13837775	13838087
chr19	2715361	2715715
chr19	11676262	11677933

chr20	63953098	63953408
chr20	62806260	62806496
chr20	62366948	62367030
chr20	63659689	63660674
chr20	63529149	63529330
chr20	38129160	38129333
chr22	42557273	42557585
chr22	42564427	42564730
chr22	42567747	42568029
chr22	42559799	42560114
chr22	42571478	42571721
chr22	42572751	42572969
chr22	42568690	42568922
chr22	42573243	42573559
chr22	42567382	42567700
chr22	42571722	42572117
chr22	42555410	42555509
chr22	42570794	42570947
chrX	104826133	104829583
chrX	104672099	104678122
chrX	104904301	104910378
chrX	105041515	105043241
chrX	85320258	85323630
chrX	105029253	105035391
chrX	105073045	105079149
chrX	104846199	104848241
chrX	104889804	104895934
chrX	105684062	105690225
chrX	104602037	104608348

Supplemental Table S6. Mobile elements with expression changes that overlap regulatory regions.

chr	start	end	Regulatory Region	Status in Mammary Tissue	Mobile Element
1	108535430	108535937	promoter_flanking_region	Inactive	SVA_D:SVA:Retroposon
1	108536345	108536526	open_chromatin_region	Inactive	SVA_D:SVA:Retroposon
1	109177402	109178600	promoter_flanking_region	Inactive	SVA_D:SVA:Retroposon
2	94497425	94497666	open_chromatin_region	Inactive	L1PA2:L1:LINE
2	94498792	94498999	open_chromatin_region	Inactive	L1PA2:L1:LINE
2	99765202	99778000	promoter_flanking_region	Poised	L1PA11:L1:LINE
2	99874001	99874200	CTCF_binding_site	Repressed	AluY:Alu:SINE
2	99927402	99929999	promoter_flanking_region	Active	AluSp:Alu:SINE
2	99984601	99987800	promoter_flanking_region	Inactive	AluY:Alu:SINE
2	100057201	100057800	enhancer	Inactive	AluSx1:Alu:SINE
2	100059202	100061600	promoter_flanking_region	Inactive	AluSx1:Alu:SINE
2	101096602	101098800	promoter_flanking_region	Inactive	L1PA8A:L1:LINE
2	131624401	131624600	CTCF_binding_site	Inactive	AluY:Alu:SINE
2	131633001	131633200	CTCF_binding_site	Inactive	L1PA13:L1:LINE
2	132529264	132529453	open_chromatin_region	Inactive	L1PA4:L1:LINE
2	211889201	211889400	CTCF_binding_site	Inactive	L1PA2:L1:LINE
2	212237401	212237600	CTCF_binding_site	Repressed	L1PA7:L1:LINE
2	212237401	212238400	enhancer	Repressed	L1PA7:L1:LINE
3	23297434	23297761	open_chromatin_region	Inactive	L1PA4:L1:LINE
3	23349801	23350000	CTCF_binding_site	Inactive	L1PA3:L1:LINE
3	53366128	53366459	open_chromatin_region	Inactive	L1PA2:L1:LINE
3	53366201	53366400	CTCF_binding_site	Inactive	L1PA2:L1:LINE
3	125855069	125855323	open_chromatin_region	Repressed	L1PA4:L1:LINE
3	141759950	141760368	open_chromatin_region	Inactive	L1PA2:L1:LINE
3	141760001	141760200	CTCF_binding_site	Inactive	L1PA2:L1:LINE
3	187049801	187052400	promoter_flanking_region	Inactive	L1PA2:L1:LINE
3	187057771	187058136	open_chromatin_region	Inactive	L1PA2:L1:LINE
3	189023401	189023600	CTCF_binding_site	Repressed	L1PA3:L1:LINE
4	4019001	4019400	CTCF_binding_site	Repressed	L1PA4:L1:LINE
4	4019094	4019312	CTCF_binding_site	Repressed	L1PA4:L1:LINE
4	9080889	9081268	open_chromatin_region	Repressed	L1PA4:L1:LINE
4	39501002	39503048	promoter_flanking_region	Inactive	AluSg:Alu:SINE
4	74444600	74449401	promoter	Inactive	L1MEf:L1:LINE
4	95139362	95139648	open_chromatin_region	Inactive	L1PA5:L1:LINE
4	95139401	95139600	CTCF_binding_site	Inactive	L1PA5:L1:LINE
4	140710202	140712800	promoter_flanking_region	Inactive	AluSx3:Alu:SINE
4	150963201	150963400	CTCF_binding_site	Inactive	SVA_B:SVA:Retroposon
4	150964601	150965200	enhancer	Inactive	SVA_B:SVA:Retroposon
5	43665253	43665750	open_chromatin_region	Inactive	SVA_B:SVA:Retroposon
5	43666217	43666380	open_chromatin_region	Inactive	SVA_B:SVA:Retroposon
6	31869001	31869800	promoter_flanking_region	Inactive	AluY:Alu:SINE
6	53306001	53306200	CTCF_binding_site	Inactive	SVA_D:SVA:Retroposon
6	71360801	71361200	CTCF_binding_site	Inactive	L1PA7:L1:LINE
6	151863062	151863273	open_chromatin_region	Repressed	L1PA5:L1:LINE
6	151867475	151867710	open_chromatin_region	Inactive	L1PA5:L1:LINE

7	65480601	65481000	CTCF_binding_site	Repressed	L1PA4:L1:LINE
7	65480649	65480860	open_chromatin_region	Repressed	L1PA4:L1:LINE
8	103783445	103783667	open_chromatin_region	Repressed	L1PA2:L1:LINE
8	103795201	103797200	CTCF_binding_site	Active	L1PA5:L1:LINE
8	103795513	103795863	TF_binding_site	Inactive	L1PA5:L1:LINE
8	127934601	127939399	promoter_flanking_region	Inactive	AluSg:Alu:SINE
8	127943402	127953200	promoter_flanking_region	Active	AluY:Alu:SINE
8	128013802	128015399	promoter_flanking_region	Inactive	AluSz6:Alu:SINE
8	128057602	128071600	promoter_flanking_region	Active	AluSz:Alu:SINE
8	132674400	132676401	promoter	Inactive	AluSx:Alu:SINE
8	133496600	133500601	promoter	Inactive	AluSx1:Alu:SINE
8	133536402	133542399	promoter_flanking_region	Inactive	AluSx1:Alu:SINE
9	41155351	41155743	open_chromatin_region	Inactive	L1PA3:L1:LINE
9	63811801	63819201	promoter	Active	AluSq:Alu:SINE
9	68275557	68276003	open_chromatin_region	Inactive	L1PA3:L1:LINE
9	86160686	86160855	open_chromatin_region	Repressed	L1PA2:L1:LINE
10	77820337	77820764	open_chromatin_region	Inactive	AluSx1:Alu:SINE
12	48915168	48915389	open_chromatin_region	Repressed	SVA_D:SVA:Retroposon
12	48915564	48915780	open_chromatin_region	Repressed	SVA_D:SVA:Retroposon
12	100293647	100293844	TF_binding_site	Inactive	SVA_D:SVA:Retroposon
12	100295401	100295600	CTCF_binding_site	Inactive	SVA_D:SVA:Retroposon
13	49124401	49130600	promoter_flanking_region	INactive	L1PA7:L1:LINE
13	49161171	49161452	open_chromatin_region	Inactive	L1P2:L1:LINE
13	96331201	96331400	enhancer	Inactive	L1PA4:L1:LINE
14	65633318	65633524	TF_binding_site	Inactive	SVA_D:SVA:Retroposon
14	65634190	65634544	open_chromatin_region	Inactive	SVA_D:SVA:Retroposon
15	89195001	89200599	promoter_flanking_region	Active	SVA_F:SVA:Retroposon
15	89200801	89201000	promoter_flanking_region	Inactive	AluSq:Alu:SINE
17	42398001	42405200	promoter_flanking_region	Inactive	AluJr:Alu:SINE
17	42402801	42403800	CTCF_binding_site	Inactive	AluJr:Alu:SINE
19	44744800	44751601	promoter	Active	AluSx1:Alu:SINE
20	62802600	62807001	promoter	Inactive	AluSx1:Alu:SINE
20	63953000	63953601	promoter	Repressed	AluSp:Alu:SINE
21	8235401	8236000	CTCF_binding_site	Poised	AluSx1:Alu:SINE
21	8417801	8418800	CTCF_binding_site	Poised	AluSx1:Alu:SINE
21	8462401	8463200	CTCF_binding_site	Poised	AluSx1:Alu:SINE
X	25882401	25882800	enhancer	Repressed	L1P2:L1:LINE
X	94483001	94483200	CTCF_binding_site	Repressed	L1PA8A:L1:LINE
X	104889547	104889834	open_chromatin_region	Repressed	L1PA5:L1:LINE
X	104908822	104908966	open_chromatin_region	Repressed	L1PA2:L1:LINE
X	105034601	105035000	CTCF_binding_site	Inactive	L1PA4:L1:LINE
X	105684697	105684815	TF_binding_site	Repressed	L1PA6:L1:LINE

Supplemental Table S7. Table showing the primer sets used to amplify the L1 and *Alu* insertions identified in Patient 2.

Primer	Sequence	
Forward Primer	CCCAAAGGACTATAAATCATGCTG	L1
Reverse Primer	CAAGACCAATCTCGTGCTACT	L1
Forward Primer	GATCTCGGCTCACTGCAAG	*Alu*
Reverse Primer	AGCCACTGGGTTAGAAAGCA	*Alu*
